# Recurring Influenza B Virus Infections in Seals 

**DOI:** 10.3201/eid1903.120965

**Published:** 2013-03

**Authors:** Rogier Bodewes, Danny Morick, Gerrie de Mutsert, Nynke Osinga, Theo Bestebroer, Stefan van der Vliet, Saskia L. Smits, Thijs Kuiken, Guus F. Rimmelzwaan, Ron A.M. Fouchier, Albert D.M.E. Osterhaus

**Affiliations:** Author affiliations: Erasmus Medical Centre, Rotterdam, the Netherlands (R. Bodewes, G. de Mutsert, T. Bestebroer, S. van der Vliet, S.L. Smits, T. Kuiken, G.F. Rimmelzwaan, R.A.M. Fouchier, A.D.M.E. Osterhaus);; Seal Rehabilitation and Research Centre, Pieterburen, the Netherlands (D. Morick, N. Osinga);; ViroClinics Biosciences BV, Rotterdam (S.L. Smits, G.F. Rimmelzwaan, A.D.M.E. Osterhaus)

**Keywords:** influenza B virus, seals, serology, viruses, influenza, *Suggested citation for this article:* Bodewes R, Morick D, de Mutsert G, Osinga N, Bestebroer T, van der Vliet S, et al. Recurring influenza B virus infections in seals [letter]. Emerg Infect Dis [Internet]. 2013 Mar [*date cited*]. http://dx.doi.org/10.3201/eid1903.120965

**To the Editor:** Until 1999, influenza B virus was considered to infect humans only. However, more recent data proved that harbor seals (*Phoca vitulina*) and gray seals (*Halichoerus grypus*) also can be infected (*1*). Since the identification of seals as a novel host, antibodies against human influenza B viruses have been detected in some additional otarid and phocid species in a few relatively small studies (*2*,*3*). It has been speculated that seals may be an animal reservoir for human influenza B viruses, although whether influenza B viruses continues to circulate among pinnipeds is unknown.

To investigate whether influenza B viruses had continued to circulate in seals, we analyzed serum samples from 615 seals (548 harbor seals [*Phoca vitulina*] and 67 gray seals [*Halichoerus grypus*]). The samples had been collected upon the animal’s admission to the Seal Rehabilitation and Research Centre (SRRC) in Pieterburen, the Netherlands, from seals living in Dutch coastal waters during 2002–2012. We tested these samples for influenza B virus–specific antibodies with a previously described hemagglutination inhbition (HI) assay, using the following influenza B virus strains as antigens: B/Seal/Netherlands/1/1999, B/Jiangsu/010/2003, B/Yamanashi/166/1998, and B/Malaysia/2506/2004 (*4*). Influenza B virus–specific antibodies were not detected in serum specimens collected from seals during 2002–2009 and after 2011; however, in 2010, HI serum antibodies against influenza B viruses were detected in 9 of 21 samples, and in 2011, they were detected in 1 of 150 samples from both harbor seals (n = 6) and gray seals (n = 4) (Figure, panel A). Nine of these positive samples were collected from juvenile seals 6–12 months of age with severe respiratory disease, and 1 was collected from a pup of ≈4 weeks of age. In seals >6 months of age, maternal antibodies have declined to undetectable levels (*5*). Therefore, these 9 juvenile seals must have become infected from late 2009 through early 2010. This suggests that the infection was caused by the novel introduction of an influenza B virus in seals in the coastal waters of the Netherlands, either by seals or by another source. Because most serum samples were collected within 1 day of the animal’s arrival at SRRC, seals must have been infected in the wild and not at the center.

Although the 9 positive samples found in 2010 represent 43% of the tested serum samples for that year, this finding does not reflect the proportion of seropositive seals in the population. Only a limited number of seals of the population, most with respiratory problems, are admitted to SRRC, and serum is not collected from all these animals.

Although the 9 seropositive seals, all >6 months of age, had been admitted to SRRC with severe respiratory signs, it should be noted that severe respiratory disease in seals has many other causes (*6*). Because no respiratory samples suitable for diagnostic purposes had been stored, the viral agent could not be determined. Consequently, whether the influenza B virus infection of these seals, as evidenced by serologic test results, had been symptomatic could not be ascertained.

To further characterize the influenza B virus strain that most likely had infected the seropositive seals, we tested their serum samples for the presence of HI antibodies against additional human influenza B virus antigens of both B/Yamagata/16/88 and B/Victoria/2/87 lineages. These represent influenza B viruses that circulated in humans during the past 20 years. Highest mean antibody titers were measured against influenza B/Yamanashi/166/98 (mean HI titer 781, SD 168), whereas lower antibody titers were detected against all other viruses, including influenza B/Seal/Netherlands/1/99, which was isolated from seals in the Dutch coastal waters in 1999 (Figure, panel B). These results suggest that the seals had been infected with an influenza B virus similar to B/Yamanashi/166/98, which is antigenically different from B/Seal/Netherlands/1/1999. 

On the basis of serum antibody titers against influenza B virus antigens tested and those of respective homologs of ferret antiserum samples, an antigenic map was prepared as described (*7*). This map, in which the antigenic distance between serum and antigen is inversely correlated with the HI titer, shows relative positions of the antigens and serum samples. Also in this map, tested seal serum samples appear to be most closely associated with influenza B/Yamanashi/166/98 **(**Figure, panel C). The obvious remaining question is whether the influenza B virus that had infected these seals in 2009–2010 is a drifted variant of influenza B/Seal/1/99 or the result of another introduction of a human influenza B virus several years ago. No evidence for transmission of influenza B virus from seals to humans was found when strains circulating in humans in the Netherlands were compared with those circulating in seals during the observation period (*8*,*9*). In conclusion, results of this study confirm that influenza B viruses continue to infect seals and support the notion that seals could serve as a reservoir of human influenza B viruses. 

**Figure:**
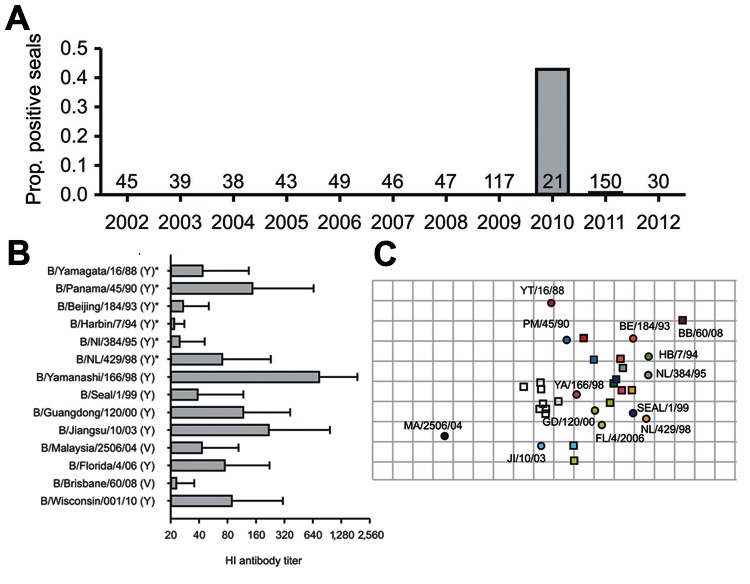
Serologic evidence of influenza B virus in seals, the Netherlands, 2002–2012. A) Proportion (prop.) of serum samples for each year shown that were positive for influenza B virus antibodies. The number above the year represents the serum samples tested for that year. B) Mean hemagglutination inhibition (HI) antibody titers (±SD) of tested positive serum samples against different influenza B strains belonging to either the Yamagata (Y) or Victoria lineage (V). Asterisk indicates that only 8 of 10 serum samples were tested against these strains because of the limited amount of serum available from 2 seals. C) Antigenic map of influenza B viruses. Indicated are the relative positions of strains (circles) and ferret antiserum samples (squares). Symbols of strains and homologs of ferret antiserum samples are identical, whereas serum samples of seals are indicated as unfilled squares. The spacing between grid lines is 1 antigenic unit, which corresponds to a 2-fold dilution of antiserum in the hemagglutination inhibition assay. Only serum samples and antigens that were positive against at least 3 influenza B virus antigens or serum samples were included in the antigenic map. YT, Yamagata; PM, Panama; BE, Beijing: BB, Brisbane; HB, Harbin; NL, Netherlands; YA, Yamanashi; GD, Guandong; MA, Malaysia; FL, Florida; JI, Jiangsu
